# Supplementary material for: Modeling glioblastoma heterogeneity as a dynamic network of cell states
Source: Mol Syst Biol. 2021 Sep 16;17(9):e10105. doi: 10.15252/msb.202010105 (PMC8444284; doi:10.15252/msb.202010105)
Supplement: Supplementary file 5 — Source Data for Figure 3 [file MSB-17-e10105-s001.zip › Figure3A_sourcedata/GSEA_3065/hallmarks_state3.GseaPreranked.1623416274744/HALLMARK_IL2_STAT5_SIGNALING.html]

Details for gene set HALLMARK\_IL2\_STAT5\_SIGNALING[GSEA]

|  || Dataset | state3 |
| Phenotype | NoPhenotypeAvailable |
| Upregulated in class | na\_pos |
| GeneSet | HALLMARK\_IL2\_STAT5\_SIGNALING |
| Enrichment Score (ES) | 0.32204053 |
| Normalized Enrichment Score (NES) | 1.2645904 |
| Nominal p-value | 0.053412464 |
| FDR q-value | 0.14708488 |
| FWER p-Value | 0.808 |
Table: GSEA Results Summary

  

Fig 1: Enrichment plot: HALLMARK\_IL2\_STAT5\_SIGNALING      
 Profile of the Running ES Score & Positions of GeneSet Members on the Rank Ordered List

  

| PROBE | GENE SYMBOL | GENE\_TITLE | RANK IN GENE LIST | RANK METRIC SCORE | RUNNING ES | CORE ENRICHMENT || 1 | ALCAM |  |  | 45 | 0.440 | 0.0328 | Yes |
| 2 | WLS |  |  | 58 | 0.407 | 0.0665 | Yes |
| 3 | CD81 |  |  | 109 | 0.337 | 0.0898 | Yes |
| 4 | SYT11 |  |  | 111 | 0.335 | 0.1186 | Yes |
| 5 | COL6A1 |  |  | 254 | 0.261 | 0.1247 | Yes |
| 6 | PHTF2 |  |  | 292 | 0.245 | 0.1416 | Yes |
| 7 | ITGAV |  |  | 322 | 0.238 | 0.1587 | Yes |
| 8 | IGF2R |  |  | 348 | 0.230 | 0.1757 | Yes |
| 9 | HUWE1 |  |  | 388 | 0.221 | 0.1903 | Yes |
| 10 | PLEC |  |  | 461 | 0.207 | 0.1998 | Yes |
| 11 | ITGA6 |  |  | 502 | 0.198 | 0.2123 | Yes |
| 12 | NT5E |  |  | 511 | 0.197 | 0.2284 | Yes |
| 13 | HIPK2 |  |  | 545 | 0.193 | 0.2412 | Yes |
| 14 | PRNP |  |  | 609 | 0.185 | 0.2499 | Yes |
| 15 | P4HA1 |  |  | 649 | 0.179 | 0.2608 | Yes |
| 16 | IFNGR1 |  |  | 733 | 0.166 | 0.2655 | Yes |
| 17 | PTCH1 |  |  | 743 | 0.165 | 0.2788 | Yes |
| 18 | LRIG1 |  |  | 772 | 0.161 | 0.2894 | Yes |
| 19 | SNX14 |  |  | 789 | 0.159 | 0.3012 | Yes |
| 20 | TWSG1 |  |  | 893 | 0.149 | 0.3022 | Yes |
| 21 | AHNAK |  |  | 923 | 0.146 | 0.3114 | Yes |
| 22 | SLC2A3 |  |  | 1026 | 0.136 | 0.3114 | Yes |
| 23 | XBP1 |  |  | 1036 | 0.135 | 0.3220 | Yes |
| 24 | CYFIP1 |  |  | 1224 | 0.120 | 0.3108 | No |
| 25 | CCND2 |  |  | 1286 | 0.116 | 0.3137 | No |
| 26 | SPP1 |  |  | 1358 | 0.111 | 0.3151 | No |
| 27 | SPRY4 |  |  | 1461 | 0.105 | 0.3124 | No |
| 28 | EMP1 |  |  | 1624 | 0.096 | 0.3019 | No |
| 29 | CKAP4 |  |  | 1727 | 0.090 | 0.2979 | No |
| 30 | CD44 |  |  | 1748 | 0.089 | 0.3033 | No |
| 31 | POU2F1 |  |  | 1978 | 0.077 | 0.2835 | No |
| 32 | ENPP1 |  |  | 1997 | 0.076 | 0.2880 | No |
| 33 | IGF1R |  |  | 2037 | 0.074 | 0.2899 | No |
| 34 | LCLAT1 |  |  | 2053 | 0.074 | 0.2945 | No |
| 35 | MYC |  |  | 2324 | 0.064 | 0.2688 | No |
| 36 | BMPR2 |  |  | 2559 | 0.055 | 0.2465 | No |
| 37 | SNX9 |  |  | 2826 | 0.046 | 0.2197 | No |
| 38 | SWAP70 |  |  | 2839 | 0.046 | 0.2223 | No |
| 39 | CSF1 |  |  | 3363 | 0.031 | 0.1645 | No |
| 40 | APLP1 |  |  | 3403 | 0.030 | 0.1627 | No |
| 41 | IFITM3 |  |  | 3449 | 0.029 | 0.1600 | No |
| 42 | SYNGR2 |  |  | 3636 | 0.025 | 0.1406 | No |
| 43 | SLC39A8 |  |  | 3655 | 0.024 | 0.1406 | No |
| 44 | ADAM19 |  |  | 3674 | 0.024 | 0.1406 | No |
| 45 | PTRH2 |  |  | 3731 | 0.022 | 0.1360 | No |
| 46 | FAM126B |  |  | 3775 | 0.021 | 0.1328 | No |
| 47 | AHR |  |  | 4020 | 0.015 | 0.1059 | No |
| 48 | CDC6 |  |  | 4028 | 0.014 | 0.1063 | No |
| 49 | NCOA3 |  |  | 4125 | 0.012 | 0.0962 | No |
| 50 | PDCD2L |  |  | 4267 | 0.008 | 0.0806 | No |
| 51 | PLSCR1 |  |  | 4373 | 0.005 | 0.0688 | No |
| 52 | EEF1AKMT1 |  |  | 4413 | 0.004 | 0.0646 | No |
| 53 | AHCY |  |  | 4459 | 0.002 | 0.0596 | No |
| 54 | UMPS |  |  | 4508 | 0.001 | 0.0542 | No |
| 55 | PLPP1 |  |  | 4721 | -0.004 | 0.0300 | No |
| 56 | CDKN1C |  |  | 4860 | -0.007 | 0.0147 | No |
| 57 | UCK2 |  |  | 5042 | -0.012 | -0.0052 | No |
| 58 | CA2 |  |  | 5274 | -0.018 | -0.0303 | No |
| 59 | BHLHE40 |  |  | 5331 | -0.019 | -0.0351 | No |
| 60 | MYO1C |  |  | 5425 | -0.022 | -0.0440 | No |
| 61 | KLF6 |  |  | 5627 | -0.028 | -0.0648 | No |
| 62 | CCNE1 |  |  | 5730 | -0.031 | -0.0740 | No |
| 63 | PLIN2 |  |  | 5856 | -0.034 | -0.0855 | No |
| 64 | TIAM1 |  |  | 5861 | -0.035 | -0.0829 | No |
| 65 | GABARAPL1 |  |  | 5976 | -0.039 | -0.0928 | No |
| 66 | FURIN |  |  | 5987 | -0.039 | -0.0906 | No |
| 67 | NDRG1 |  |  | 6040 | -0.041 | -0.0930 | No |
| 68 | MAP6 |  |  | 6061 | -0.042 | -0.0918 | No |
| 69 | FAH |  |  | 6077 | -0.042 | -0.0899 | No |
| 70 | SPRED2 |  |  | 6157 | -0.045 | -0.0951 | No |
| 71 | BMP2 |  |  | 6331 | -0.050 | -0.1108 | No |
| 72 | ST3GAL4 |  |  | 6391 | -0.052 | -0.1131 | No |
| 73 | SERPINB6 |  |  | 6404 | -0.053 | -0.1099 | No |
| 74 | DENND5A |  |  | 6405 | -0.053 | -0.1054 | No |
| 75 | GSTO1 |  |  | 6489 | -0.056 | -0.1101 | No |
| 76 | RNH1 |  |  | 6509 | -0.057 | -0.1074 | No |
| 77 | CDC42SE2 |  |  | 6587 | -0.060 | -0.1111 | No |
| 78 | MAPKAPK2 |  |  | 6613 | -0.062 | -0.1087 | No |
| 79 | GADD45B |  |  | 6619 | -0.062 | -0.1039 | No |
| 80 | ETV4 |  |  | 6660 | -0.064 | -0.1030 | No |
| 81 | RHOB |  |  | 6706 | -0.066 | -0.1025 | No |
| 82 | SOCS2 |  |  | 6715 | -0.067 | -0.0977 | No |
| 83 | BCL2L1 |  |  | 6793 | -0.071 | -0.1005 | No |
| 84 | CCND3 |  |  | 6959 | -0.079 | -0.1128 | No |
| 85 | CASP3 |  |  | 6969 | -0.079 | -0.1070 | No |
| 86 | RRAGD |  |  | 7087 | -0.086 | -0.1131 | No |
| 87 | PRAF2 |  |  | 7143 | -0.090 | -0.1117 | No |
| 88 | DCPS |  |  | 7339 | -0.104 | -0.1253 | No |
| 89 | RGS16 |  |  | 7541 | -0.122 | -0.1380 | No |
| 90 | BATF3 |  |  | 7666 | -0.135 | -0.1407 | No |
| 91 | CAPG |  |  | 7767 | -0.148 | -0.1395 | No |
| 92 | TNFRSF21 |  |  | 7822 | -0.155 | -0.1324 | No |
| 93 | GLIPR2 |  |  | 7823 | -0.155 | -0.1190 | No |
| 94 | ARL4A |  |  | 7829 | -0.155 | -0.1062 | No |
| 95 | ANXA4 |  |  | 7847 | -0.158 | -0.0945 | No |
| 96 | NCS1 |  |  | 8039 | -0.191 | -0.1001 | No |
| 97 | HOPX |  |  | 8211 | -0.231 | -0.0999 | No |
| 98 | ITGAE |  |  | 8470 | -0.325 | -0.1017 | No |
| 99 | ODC1 |  |  | 8576 | -0.385 | -0.0806 | No |
| 100 | PHLDA1 |  |  | 8670 | -0.476 | -0.0503 | No |
| 101 | GPX4 |  |  | 8742 | -0.690 | 0.0010 | No |
Table: GSEA details [plain text format]

  

Fig 2: HALLMARK\_IL2\_STAT5\_SIGNALING: Random ES distribution      
 Gene set null distribution of ES for **HALLMARK\_IL2\_STAT5\_SIGNALING**
